# Infective Endocarditis After Renal Transplantation

**DOI:** 10.5812/numonthly.12326

**Published:** 2014-01-13

**Authors:** Maryam Moshkani Farahani, Zohreh Rostami, Behzad Einollahi, Arezoo Khosravi, Eghlim Nemati, Mahboob Lessan Pezeshki, Vahid Pourfarziani, Nematollah Joneidi, Mohammad Javad Hosseini, Gholam Ali Ghorbani

**Affiliations:** 1 Cardiology Research Center, Baqiyatallah University of Medical Sciences, Tehran, IR Iran; 2 Nephrology and Urology Research Center, Baqiyatallah University of Medical Sciences, Tehran, IR Iran; 3 Departement of Nephrology, Tehran University of Medical Sciences, Tehran, IR Iran; 4 Molecular Biology Research Center, Baqiyatallah University of Medical Sciences, Tehran, IR Iran

**Keywords:** Endocarditis, Kidney Transplantation, Infection

## Abstract

**Background:**

Infective endocarditis (IE) is a serious complication in immunosuppressive patients that has adverse effects.

**Objectives:**

The aim of this study was to define the characteristics, outcomes, and correlating factors of mortality in renal transplant recipients.

**Patients and Methods:**

Infective endocarditis was diagnosed in 22 patients from three renal transplant centers in Iran between 2000 and 2010. Modified Duke criteria were applied to confirm the diagnosis.

**Results:**

Twenty-two renal transplant patients with IE were evaluated. Blood culture results were positive in 81%. *Enteroccous* and group D non-enterococcal were the causative microorganisms in 31% and 25% of patients, respectively. In-hospital and 12-month mortality was 41% and the mortality rate was higher in older patients in comparison to younger patients. Overall, the rates of one-year disease-free patient and graft survival were 49% and 88%, respectively.

**Conclusions:**

Despite the availability of different and potent antibiotics, the mortality caused by IE remains considerably high. These patients are significantly prone to endovascular infections that affect the mortality and survival.

## 1. Background

In recent years, the number of patients undergoing renal transplantation has significantly increased. Prolonged duration of corticosteroids consumption, graft failure which requires hemodialysis, and potent immunosuppressive therapy are supposed to put these patients at the risk of serious infections after renal transplantation. Infective endocarditis (IE) is one of the important and serious infections that affect these patients. Kidney transplant patients are susceptible to IE, which increases graft loss and mortality rate (1). Immunosuppressive therapy is the most important predisposing factor. Moreover, intensification of immunosuppressant to treat renal allograft rejection poses an additional risk factor for IE. On the other hand, decreased or withdrawal of immunosuppressive therapy broughts the risk of rejection. Endocarditis is substantially more common in renal transplant patients than in general populations. In developed countries, the incidence of IE ranges from 3.6 to 7 cases per 100000 populations per year (2, 3). The incidence rates of IE per 1000 patient has been reported 2.6 among deceased-donor transplant recipients and 1.8 among living-donor transplant recipients (4). Prompt diagnosis and treatment is mandatory because of significant complication and poor outcome. In other words, high index of suspicion must be maintained for diagnosis of IE due to different clinical features, especially in patients with immunosuppression therapy. Renal transplant patients are predisposed to developing IE, which is associated with poor long-term survival (4).

## 2. Objectives

Infective endocarditis is a known complication of kidney transplantation. However, published information has been limited to case reports and small case series. Although the rate of kidney transplantation in Iran is high, only a few published works on incidence of IE in Iranian kidney transplants are currently available (1, 5). Thus, we made a plan to determine the impact of IE on graft and patient outcomes in renal transplant recipients.

## 3. Patients and Methods

In a retrospective study, all patients with kidney transplant complicated with IE from three renal transplant centers in Tehran, Iran between 2000 and 2010 were enrolled. Demographic, clinical, echocardiographical and microbiological data were obtained.

Demographic and clinical data included age, sex, donor source (living related or unrelated versus deceased donor), immunosuppressant regimen, clinical presentation, time presentation since renal transplantation, co-infections such as CMV infection, UTI and other infections, type of treatment, response to therapy, organisms responsible for causing IE and echocardiographic findings as well as short term (6 months) patient and graft survival after diagnosis of IE and in-hospital mortality.

All patients underwent echocardiographic evaluation. All of patients were examined by both transthoracic echocardiography (TTE) and transesophageal echocardiography (TEE). Diagnosis of IE was based on modified Duke criteria. According to modified Duke criteria, definite IE is based on obtaining pathologic microorganism by culture or histology or pathological lesion like vegetation, intracardiac abscess and clinical criteria (3).

## 3.1. Statistical Analysis

Data were analyzed using SPSS for Windows version 17.0. The quantitative results were presented as mean SD, while qualitative variables were expressed by number and percentage. Continuous data were compared by Mann-Whitney test. Categorical data were analyzed using the Chi-square or Fisher’s exact test. Overall one year free disease patient and graft survival rates after diagnosis of IE were calculated using the Kaplan-Meier method. The Log-rank test was used to determine statistical differences in graft survival between patients with and without CMV infection. A result was considered statistically significant when the p-value was less than 0.05.

## 4. Results

In the period of 10 years (2000-2010) study, we found twenty-two kidney transplants with IE. The mean age of patients was 47 ± 18 years (range: 19-72 years; median: 39.5 years). All the 22 patients, 12 male and 10 female, had renal transplantation for the first time but one patient received a kidney for second transplantation. The kidney was harvested from a living unrelated donors and deceased donors in 17 and five patients, respectively. The mean follow up period was 14 ± 18 months.

Immunosuppressive regimen was based on cyclosporine, myophenolate mofetil (MMF), and prednisolone in 20 cases and two cases received cyclosporine, azathioprine, and prednisolone. Two patients had history of anti-thymocyte globulin (ATG) therapy and two cases had history of steroid pulse therapy.

The mean time of IE presentation since renal transplantation was 21 ± 36 months (2-120 months, median = 5.5 months). The most frequent clinical presentations of IE were fever in 100%, anemia in 73% and weakness in 59% of patients.

Blood culture results were positive in 81% of cases. Enteroccous and group D non-enterococcal were the causative microorganisms in 31% and 25% of patients, respectively. Other cultured organisms are shown in Table 1. Co-infection with CMV was seen in 43% of patients. Other diagnosed infections are listed in Table 2. 

**Table 1.** Prevalence of Microorganism Pathogens

Microorganism	Culture Result, %
***Enterococcus***	31
**Group D non-enterococcal**	25
***Staph aureus***	12.5
***Kelebsiella***	6.25
***Streptococcus***	6.25
**Negative blood culture **	19

**Table 2.** Different Types of Infections in Renal Transplant Patients With IE^a^

Type of Infection	Affected Cases, No.
**Sepsis**	10
**Endophthalmitis**	1
**Meningitis**	1
**UTI** ^**a**^	6
**Liver abscess**	1
**Brain abscess**	7

^a^ Abbreviations: IE, Infective endocarditis; UTI, urinary tract infection.

There was no evidence of vegetation in 7 cases by TTE (i.e. negative TTE), but TEE was diagnostic and showed vegetation in all patients. Nineteen percent of patients had negative blood culture results; however, their echocardiographic findings were consistent with vegetations that fulfilled the criteria of duke for IE diagnosis. Multiple vegetations (involving more than one valve) were seen in 15% of cases and two valves involvement was seen in 10% and one cardiac valve involvement was detected in 75% patients.

The aortic valve was the most frequently affected valve and was seen in 50% patients. The mitral valve and tricuspid valve involvement was seen in 45.5% and 4.5% of patients, respectively. Mean size of vegetation was 1.22 ± 0.49 cm (0.5-2.2 cm, median = 1.1 cm). Six patients underwent cardiac surgery, but two of them died. In patients with CMV infection, IE was occurred earlier after renal transplantation (4 ± 1 months vs. 36 ± 45 months, P = 0.01).

There was no significant correlation between death and presenting illness (P = 0.5) as well as with vegetation size (P = 0.3). No significant association was seen between death of patients and CMV infection (P = 0.6).

All patients with IE were treated by antibiotics along with decrease or withdrawal of immunosuppressive therapy with or without cardiac surgery. Acute rejection due to reduction or withdrawal of immunosuppressive therapy was only seen in 12% of patients. Allograft renal function remained within normal range in 75%, graft failure (reduction of GFR) was seen in 15% and graft loss occurred in 10% of recipients with IE.

Fourteen patients (64%) responded to treatment. In-hospital and 12 months mortality was 41%. Mortality rate was higher in older patients compared to younger cases (7 cases were 48 years old or older). The mean age of deceased recipients was 52 years old and the mean age of alive patients was 42 years old (P = 0.2).

Six-month graft survival after IE diagnosis in patients with CMV infection was 88%, while it was 100% in patients without CMV infection (Log-rank = 0.16). Six-month patient's survival after IE diagnosis was 68%. No significant difference was seen between CMV infected patient and those without CMV infection (Log-rank = 0.25). There were no significant differences in short-term patient and graft survival between cases with and without CMV infection.

One-year patient survival rates were 60% in male and 47% in female, respectively (P = 0.08). In patients younger than 35 and older than 35 years of age, the rate was 83% and 36%, respectively (P = 0.04). Overall one year free disease patient and graft survivals were 49% and 88%, respectively.

## 5. Discussion

Renal transplant patients are prone to many infections like other organ transplant patients because of administration of immunosuppressive therapy (6, 7). Kidney transplant patients complicated with IE need considerable attention because of its higher rate of mortality and complications. In this study, our experience of renal transplant patients with IE is outlined. Our findings confirmed that IE was common after kidney transplantation. Similar to other studies, our result showed that in contrast to general population, organ transplantation is associated with high risk of endocarditis (6, 8).

*Enterococcus* and group D non-streptococcus bacteria remain the most common pathogen in this group that accounting for 30% and 25% of all IE. This is consistent with other studies results (5, 9). Conversely, in the study of Baroudi et al. the prevalence of pathogenic microorganisms were different and *Staphylococcus aureus* and streptococcal species were responsible for IE in 29% and 33% of patients, respectively (4).

In consistent with other studies, the mitral and the aortic valves were the most affected valves than right sided valves (4). TEE with 85-95% sensitivity (10) played an important role for detecting vegetations that were not diagnosed by TTE. The size of vegetations on the valves of heart were moderate to large in majority of patients with the mean vegetation size of 1.2 cm (0.5-2.2 cm). 

In our study, in-hospital and 12 months mortality was 41% with higher rate of mortality in elderly patients (7 cases ≥ 48 years); while in other studies, the mortality of IE in transplant recipients has been reported to be 44-57% (4, 7, 9). This is reasonable due to weaker immune system in older patients. However, limited data are currently available in literature about long-term survival in these patients. In addition, poor patient survival rate among kidney transplants with IE has also been demonstrated in other studies (4, 9). Our study also showed poor one-year survival after diagnosis of IE; overall one year disease-free patient and graft survival were 49% and 88%, respectively. In the study of Shroff et al., 2-year survival after IE was about 58% (4). Although in this era different and potent antibiotics are available, the mortality rate of IE remains considerably high and their survival after IE has changed slightly during recent years. On the other hand, Shroff et al. showed a higher in-hospital mortality rate in patients with IE who were on waiting list in comparison to kidney transplants complicated with IE (18.6% versus 16%) (4). In our patients, acute rejection was only seen in 12% of patients while renal function remained within normal range in 75% of patients.

Although IE is more likely to be seen in male recipients (7), there was no significant difference in IE frequency between genders in our patients. However, one year patient survival was higher in male than female with no statistically significant difference in the current study.

Simillar to other studies (6), septicemia was a common problem that was observed in 45% of recipients in our study. In addition, immunosuppression therapy in kidney transplant patients may lead to increase rate of co-infections such as CMV infection (43%) and endophthalmitis, meningitis, urinary tract infections, liver and brain abscess, which were seen in our patients.

Renal transplant patients have high in-hospital and one-year mortality rates. It is necessary to have strategies for preventing and reducing the rate of IE in this susceptible group.
